# Supplementary material for: The European Institute of Oncology Thyroid Imaging Reporting and Data System for Classification of Thyroid Nodules: A Prospective Study
Source: J Clin Med. 2022 Jun 6;11(11):3238. doi: 10.3390/jcm11113238 (PMC9181754; doi:10.3390/jcm11113238)
Supplement: Supplementary file 1 [file jcm-11-03238-s001.zip › jcm-1647807-supplementary.pdf]

Supplementary Material

Table S1. Sensitivity, specificity and diagnostic accuracy according to different cutoff of IEO-TIRADS score.

Cutoff	Diagnostic Performance		
	**Sensitivity [95% CI]**	**Specificity [95% CI]**	**Accuracy [95% CI]**
≥2 (Unlikely)	98.9% [96.8% - 100%]	16.3% [6.0% - 26.7%]	70.0% [62.4% - 77.6%]
≥3 (Doubt)	73.6% [64.6% - 82.7%]	59.2% [45.4% - 73.0%]	68.6% [60.9% - 76.3%]
≥4 (Suspected)	59.3% [49.3% - 69.4%]	79.6% [68.3% - 90.9%]	66.4% [58.6% - 74.3%]
≥5 (Malignant)	38.5% [28.5% - 48.5%]	93.9% [87.2% - 100%]	57.9% [49.7% - 66.0%]

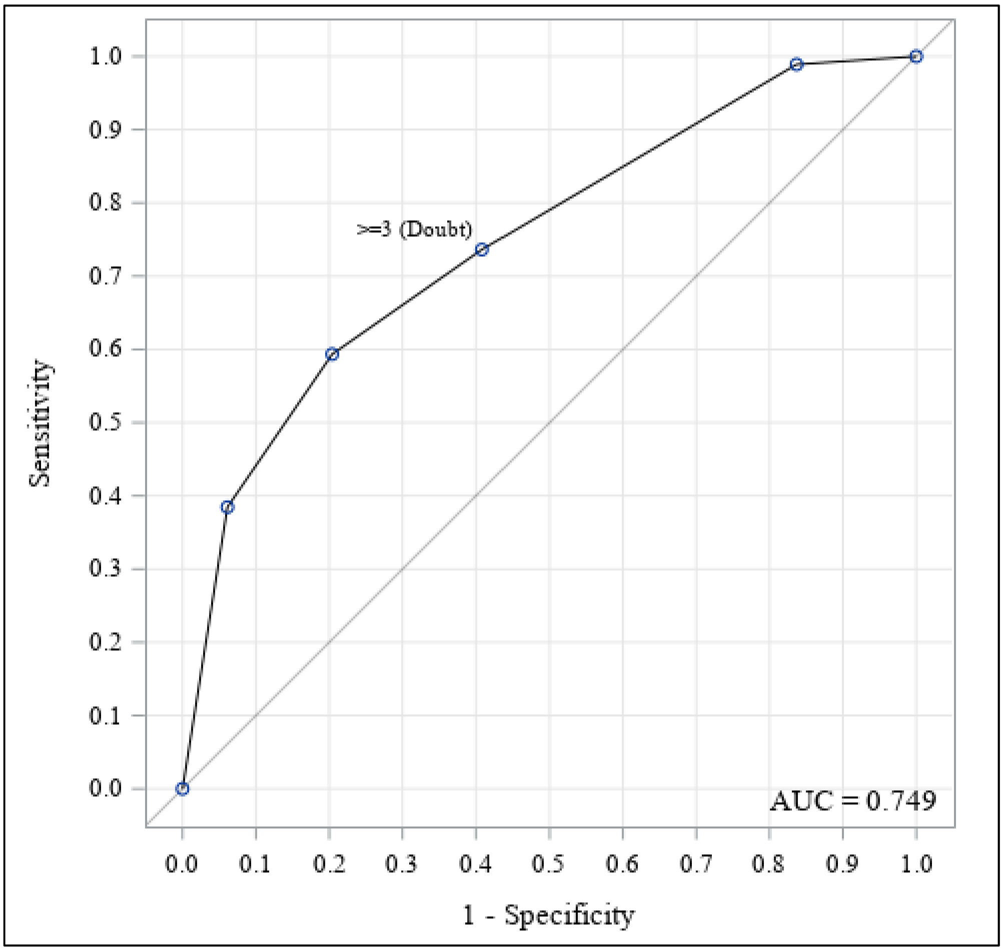

Figure S1. ROC curve of the model with IEO-TIRADS score as independent variable and histopathological result (Benign vs. Malignant) as dependent variable.
